# Brain MRI Features of Human Neuroinvasive Usutu Virus Infections

**DOI:** 10.3390/diagnostics16203269

**Published:** 2026-10-09

**Authors:** Thomas Ferenc, Tatjana Vilibić-Čavlek, Vladimir Savić, Ana Tripalo Batoš, Maja Bogdanić, Vladimir Stevanović, Dario Sabadi, Tanja Potočnik-Hunjadi, Franjo Katić, Klaudija Višković, Tonka Jozić-Novinc, Robert Škara, Ljubo Barbić, Doroteja Hunjadi, Marija Santini

**Affiliations:** 1 Department of Diagnostic and Interventional Radiology, Merkur University Hospital, 10000 Zagreb, Croatia; 2 Department of Virology, Croatian Institute of Public Health, 10000 Zagreb, Croatia; maja.bogdanic@hzjz.hr; 3 School of Medicine, University of Zagreb, 10000 Zagreb, Croatia; marijasantini.ms@gmail.com; 4 Laboratory for Virology and Serology, Poultry Center, Croatian Veterinary Institute, 10000 Zagreb, Croatia; v_savic@veinst.hr; 5 Department of Pediatric Radiology, Children’s University Hospital Zagreb, 10000 Zagreb, Croatia; abatosh@gmail.com; 6 Department of Microbiology and Infectious Diseases with Clinic, Faculty of Veterinary Medicine, University of Zagreb, 10000 Zagreb, Croatia; vladostevanovic@gmail.com (V.S.); ljubo.barbic@vef.hr (L.B.); 7 Clinic for Infectious Diseases, University Hospital Centre Osijek, 31000 Osijek, Croatia; dariocroatia@gmail.com; 8 Department of Medical Microbiology and Infectology, Faculty of Medicine, Josip Juraj Strossmayer University of Osijek, 31000 Osijek, Croatia; 9 Faculty of Dental Medicine and Health, Josip Juraj Strossmayer University of Osijek, 31000 Osijek, Croatia; 10 Clinic for Infectious Diseases, County General Hospital Varaždin, 42000 Varaždin, Croatia; tanja.potocnik.h@gmail.com; 11 Department of Diagnostic and Interventional Radiology, University Hospital Centre Osijek, 31000 Osijek, Croatia; franjo.katic@kbco.hr; 12 Department of Radiology and Ultrasound, University Hospital for Infectious Diseases Zagreb, 10000 Zagreb, Croatia; viskovick@gmail.com; 13 Faculty of Health Studies, University of Rijeka, 51000 Rijeka, Croatia; 14 School of Medicine, Catholic University of Croatia, 10000 Zagreb, Croatia; 15 Clinic for Infectious Diseases, County General Hospital “Dr. Ivo Pedišić”, 44000 Sisak, Croatia; tonkajn@gmail.com (T.J.-N.); infektologija@obs.hr (R.Š.); 16 Faculty of Veterinary Medicine, University of Zagreb, 10000 Zagreb, Croatia; dhunjadi@vef.hr; 17 Department for Infections in Immunocompromised, University Hospital for Infectious Diseases “Dr. Fran Mihaljević”, 10000 Zagreb, Croatia

**Keywords:** Usutu virus, central nervous system, magnetic resonance imaging, Croatia

## Abstract

**Background/Objectives**: Neuroradiological features of neuroinvasive Usutu virus (USUV) infection are poorly understood, as current knowledge is largely limited to case reports. This study aimed to describe brain MRI patterns, and assess disease severity and vital and functional outcomes in patients with neuroinvasive USUV infection. **Methods**: This multicentric retrospective observational cohort study analyzed adult patients of both sexes, aged ≥18 years, in the continental region of Croatia who developed symptoms of neuroinvasive disease during the arbovirus transmission season between 2017 and 2025. Patients with serological and/or molecular confirmation of USUV infection were included in further analyses. Only patients who underwent a brain MRI during hospitalization were included in the study. Clinical data and blood/CSF laboratory parameters were also evaluated. The observed outcomes were the severity of clinical symptoms, vital status at hospital discharge, and functional status at hospital discharge. **Results**: Of 214 arbovirus-positive cases, 8 patients had confirmed neuroinvasive USUV infection, and 4 had a brain MRI. Three patients were male. One patient had a normal MRI, and three patients had abnormal MRIs. All patients with abnormal MRI demonstrated meningeal contrast enhancement (a finding not previously reported), uni- or bilateral hippocampal involvement suggestive of limbic encephalitis, and basal ganglia involvement. All patients required ICU admission, experienced prolonged hospitalization, and were discharged with moderate to severe disability. **Conclusions**: This study provides important insights into MRI patterns in patients with neuroinvasive USUV infection and addresses the current knowledge gap regarding the neuroimaging of this underdiagnosed disease.

## 1. Introduction

Usutu virus (USUV) is an enveloped, single-stranded RNA arthropod-borne virus belonging to the family *Orthoflaviviridae*, genus *Flavivirus*, Japanese encephalitis serocomplex [1]. The natural cycle of USUV resembles that of West Nile virus (WNV), with birds as the main amplifying reservoir host and mosquitoes of the genus *Culex* as the primary vectors. Due to short-term and low-level viremia, humans represent incidental or dead-end hosts [1,2,3].

Human USUV infections were first described in the Central African Republic and Burkina Faso in patients presenting with fever, rash, and jaundice in 1981 and 2004, respectively [1,4]. The first cases of human neuroinvasive USUV infection in Europe were reported in two immunocompromised patients in Italy in 2009 [5,6]. Between 2012 and 2021, a total of 105 autochthonous USUV infections were detected across several European countries, including Italy [7], Austria [8], Croatia [2,9], the Czech Republic [10], France [11], Germany [12], Hungary [13], and the Netherlands [14]. The majority of cases occurred in Italy (56) and Austria (27). The number of reported human USUV infections nearly doubled from 2017 to 2021, reaching 70 cases. This increase was largely attributable to a significant rise in cases in 2018, which coincided with a surge in human WNV infections [7].

In humans, the incubation period for USUV is typically 3 to 12 days following a mosquito bite. Detecting human infections is difficult because the disease is usually asymptomatic. When symptomatic, the typical presentation includes fever, sometimes accompanied by a maculopapular rash, whereas a febrile icteric illness occurs less frequently [15]. Sporadically, the infection may evolve into a neuroinvasive form, primarily in immunocompromised patients [2], the elderly, or those with underlying comorbidities, but cases in otherwise healthy adults have also been reported [16].

USUV has been increasingly recognized as a cause of neuroinvasive disease in humans, although such cases are underreported. Patients with neuroinvasive diseases typically present with features indistinguishable from other viral meningoencephalitides caused by flaviviruses, particularly WNV [17]. Although clinical reports of USUV infection indicate involvement of brain regions similar to those affected by WNV, the clinical manifestations are generally less severe and less frequent, and do not appear to progress to the same extent as in many cases of WNV-associated neuroinvasive disease [17]. Movement disorders and cerebellar signs, including tremor, dysmetria, and ataxia, as well as cranial nerve palsies, have been documented [11,18].

In the neuroradiological workup of patients with encephalitis, magnetic resonance imaging (MRI) is preferred over computed tomography because of its superior sensitivity and specificity [19]. The neuroradiological features of neuroinvasive USUV infection remain poorly characterized, as current knowledge is limited to a small number of case reports and case series. Consequently, the spectrum of brain MRI abnormalities associated with neuroinvasive USUV infection has not yet been clearly defined.

Croatia has reported several neuroinvasive human cases, often during WNV outbreaks (2013: 3 cases; 2018: 3 cases; 2024: 5 cases) [2,3,9,20].

The aim of this study was twofold: (a) to characterize brain MRI findings among patients with neuroinvasive USUV infection in our cohort and (b) to systematically review the published literature to summarize the current reported neuroradiological manifestations of neuroinvasive USUV infection.

## 2. Materials and Methods

### 2.1. Patients

**Figure 1:**
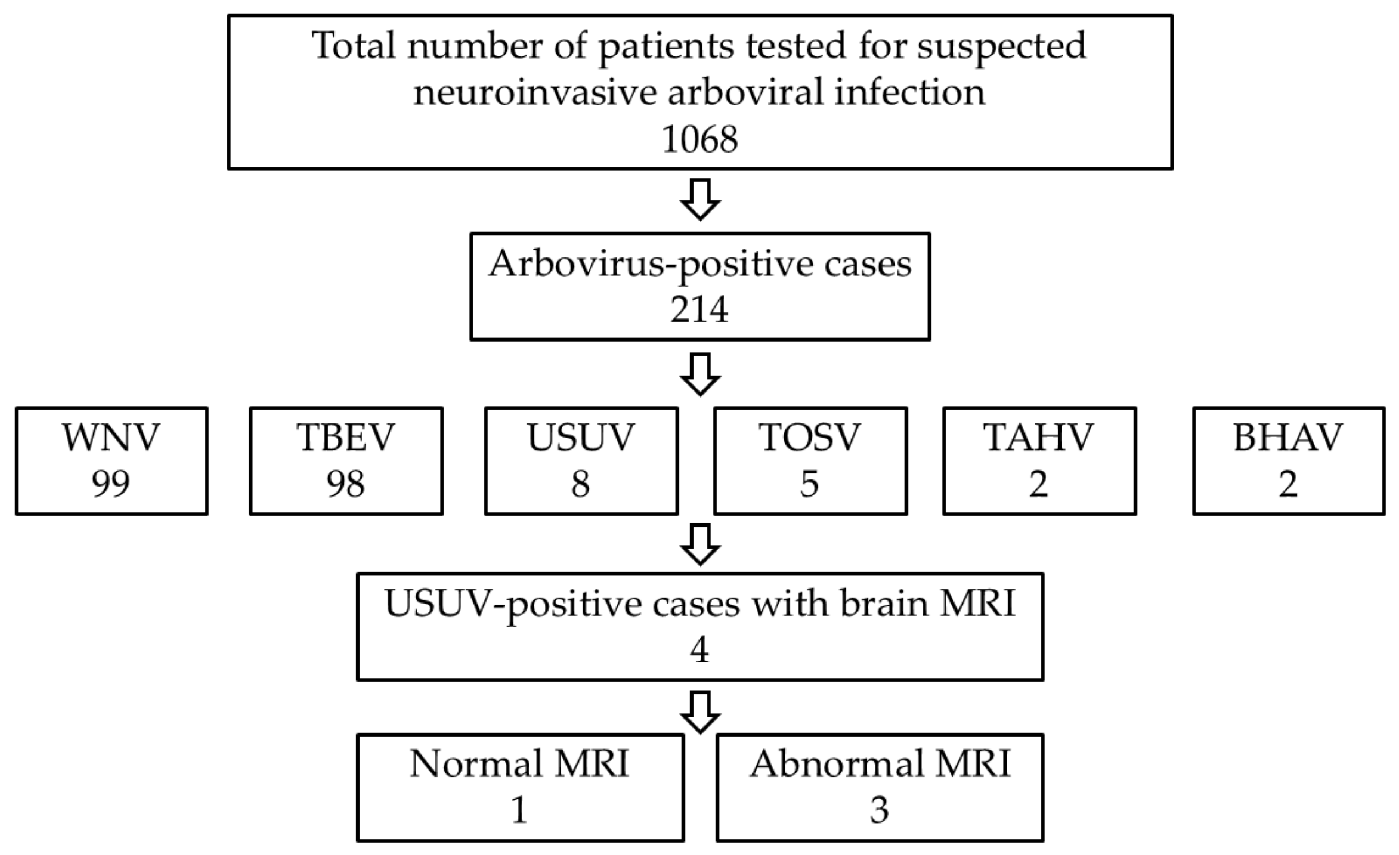
Study flow chart of patients included in the study (WNV—West Nile virus; TBEV—tick-borne encephalitis virus; USUV—Usutu virus; TOSV—Toscana virus; TAHV—Tahyna orthobunyavirus; BHAV—Bhanja bandavirus).

This multicenter retrospective observational cohort study was conducted in accordance with the Strengthening the Reporting of Observational Studies in Epidemiology (STROBE) guidelines [21]. The study population was derived from a nationwide multicenter cohort established as part of the CRONEUROARBO, FLAVIR, and CROIMUNOLAB research projects on arboviral infections in Croatia. Adult patients of both sexes (≥18 years of age) presenting with symptoms of neuroinvasive disease during the arbovirus transmission seasons between January 2017 and December 2025 were enrolled in the study. For the present analysis, patients with serologically (virus neutralization test, VNT) and/or molecularly (reverse transcription quantitative polymerase chain reaction, RT-qPCR) confirmed neuroinvasive USUV infection were identified from the parent cohort. Only patients who underwent a brain MRI during hospitalization were included in the neuroradiological analysis. Details of the patient selection process are shown in Figure 1.

### 2.2. Virological and Laboratory Testing

USUV diagnosis was confirmed according to the ECDC criteria for WNV, which included: (a) virus isolation; (b) viral RNA in blood and/or cerebrospinal fluid (CSF); (c) specific IgM antibody response in CSF; and/or (d) high IgM titer and IgG antibody detection confirmed by a virus neutralization test (VNT) [22]. Since recent studies have highlighted the utility of urine RT-qPCR for confirming WNV neuroinvasive infections, owing to its higher diagnostic sensitivity than CSF-based molecular testing, urine was included in the diagnostic evaluation for its noninvasive collection and the potential for prolonged, higher viral shedding [23,24]. In all patients, USUV infection was confirmed by detection of USUV RNA in CSF samples and by serology [24]. Clinical data and blood/CSF laboratory parameters were also evaluated.

### 2.3. Magnetic Resonance Imaging

MRI examinations were performed on 3T scanners (MAGNETOM Vida and MAGNETOM Lumina, Siemens Healthineers, Erlangen, Germany) according to standardized protocols including: T2-weighted (T2W) images (axial and coronal planes), fluid-attenuated inversion recovery (FLAIR, axial plane), and T1-weighted (T1W) images before (sagittal plane) and after gadolinium-based contrast agent administration (axial, coronal and sagittal planes), susceptibility-weighted images (SWI, axial plane), and diffusion-weighted images (DWI) with apparent diffusion coefficient (ADC) maps (axial plane). MRI examinations were independently reviewed by two radiologists: first by a general radiologist with less than 5 years of experience, and then by a neuroradiologist with more than 10 years of experience, with no prior knowledge of the original reports. Discrepancies between radiologists were resolved by consensus.

### 2.4. Outcome Evaluation

The observed outcomes were: (1) the severity of clinical symptoms, quantified as mild (0–8 points), moderate (9–22 points), or severe (≥23 points) according to Bogović et al. [25] (Table S1), (2) vital status at hospital discharge (survival or death), and (3) functional status at hospital discharge, quantified using the modified Rankin scale (mRS): 0—no symptoms at all, 1—no significant disability despite symptoms, 2—slight disability, 3—moderate disability, 4—moderately severe disability, 5—severe disability, 6—death. The severity of clinical symptoms and vital/functional status at hospital discharge were evaluated and scored by an infectious disease specialist with more than 10 years of experience in neuroinfections, who had no prior knowledge of the MRI reports.

### 2.5. Literature Review of Published Usutu Virus Cases

A literature review of PubMed/MEDLINE was conducted up to 2 September 2026 by two researchers independently (T.F. and A.T.B.) to identify confirmed human cases of neuroinvasive USUV infection with brain MRI findings. The search strategy consisted of the following keywords: (Usutu virus [Mesh] OR Usutu virus OR USUV) AND (brain magnetic resonance imaging OR brain MRI OR magnetic resonance imaging OR neuroimaging). No restrictions were applied regarding language or publication year. Eligible studies included original articles, case reports, and case series reporting confirmed neuroinvasive USUV infection with available brain MRI findings. Animal and in vitro studies, and studies without MRI data were excluded. Full texts were assessed, and reference lists of eligible studies were screened for additional reports. Disagreements were resolved by consensus. The database search identified 2 articles, and reference screening identified 4 additional eligible studies.

## 3. Results

### 3.1. Brain MRI Findings in Patients with Human Neuroinvasive Usutu Virus Infections

Of the 214 arbovirus-positive cases, 8 patients had neuroinvasive USUV infection (3 in 2018 and 5 in 2024), of whom 4 had a brain MRI (all in 2024). All patients were from the northern continental region of the country. Sociodemographic, clinical, and laboratory features of patients are presented in Table 1, and virology results are presented in Table 2.

**Figure 2:**
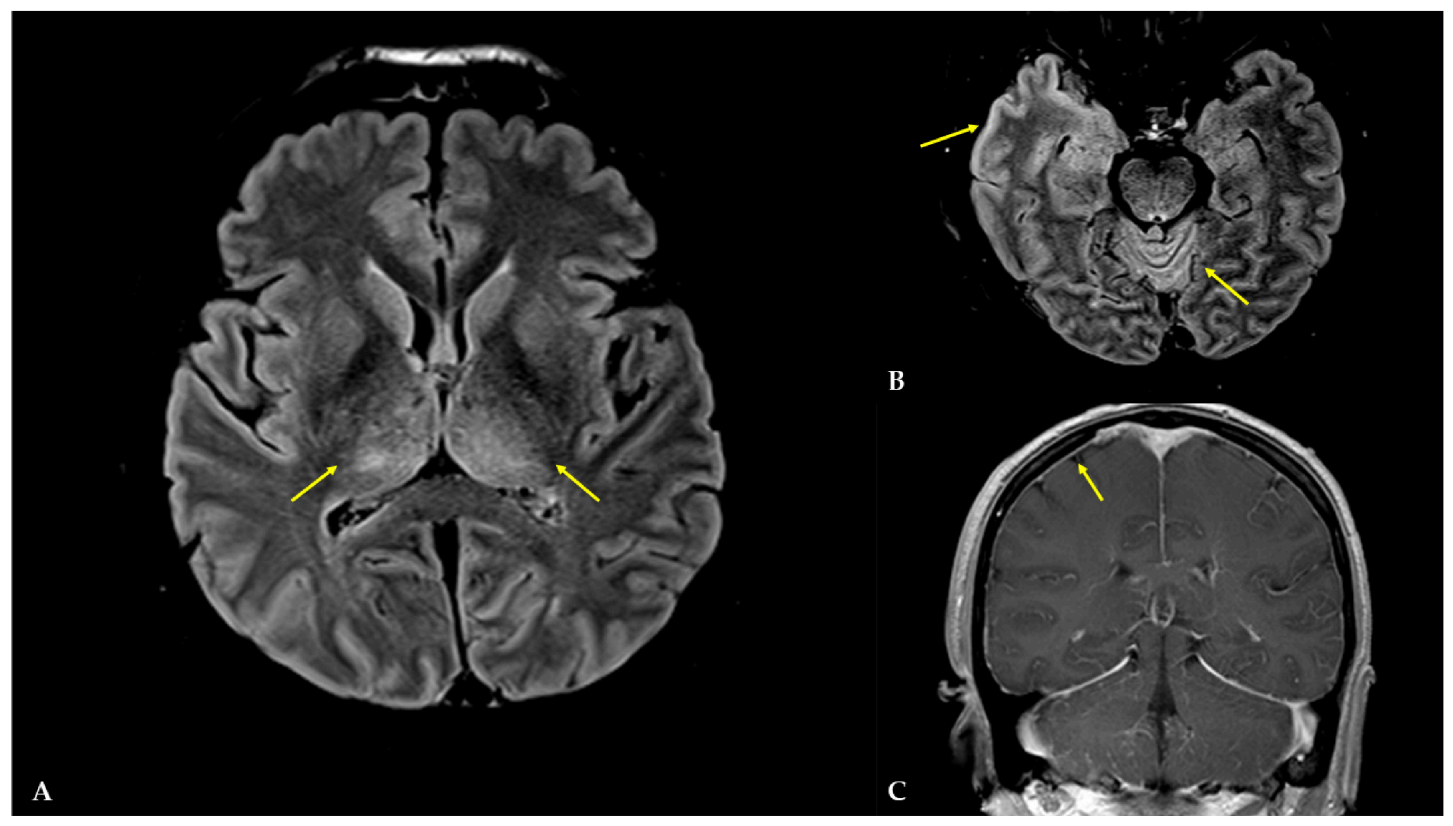
MRI images of patient 2. (**A**) Axial plane, FLAIR: asymmetric high signal intensities in both thalami (arrows); (**B**) axial plane, FLAIR: high signal intensities in the entire right temporal lobe (including hippocampus) and cerebellum (arrows); (**C**) coronal plane, T1WCE: meningeal contrast enhancement (arrows).

Patient 1 had a normal brain MRI, and three patients (Patients 2–4) had abnormal brain MRI findings. All patients with abnormal brain MRI demonstrated meningeal contrast enhancement. Patient 2 demonstrated high signal intensity in both thalami, the entire right temporal lobe (including hippocampus), and cerebellum on T2W and FLAIR images (Figure 2).

**Figure 3:**
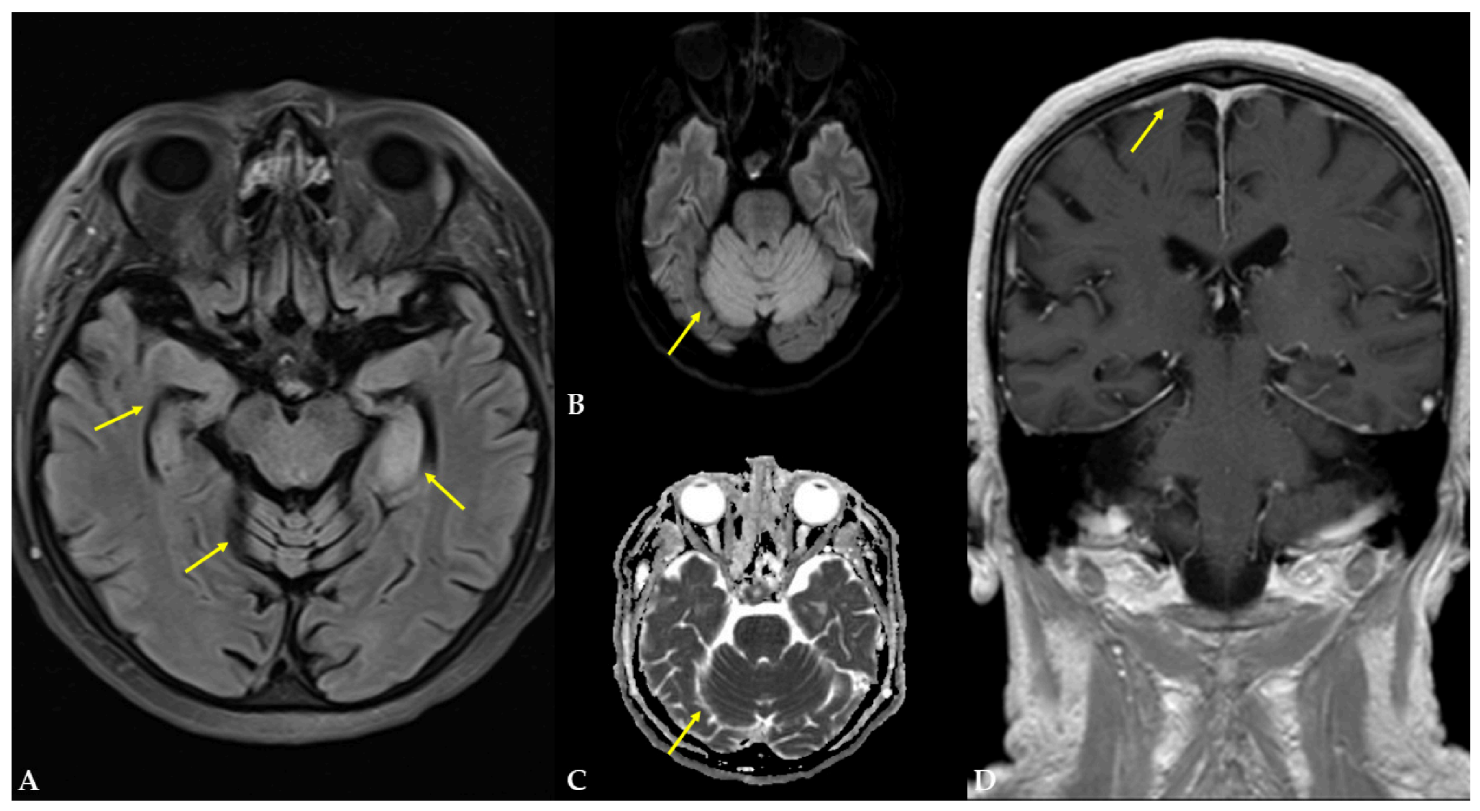
MRI images of patient 3. (**A**) Axial plane, FLAIR: asymmetric high signal intensities in both hippocampi and symmetric in cerebellar hemispheres (arrows); (**B**,**C**) axial plane, DWI/ADC: restricted diffusion in cerebellum; (**D**) coronal plane, T1WCE: meningeal contrast enhancement (arrows).

Patient 3 demonstrated asymmetric high signal intensity in both hippocampi and symmetric high signal intensity in the cerebellar hemispheres on T2W and FLAIR images, with restricted diffusion on DWI/ADC (Figure 3).

**Figure 4:**
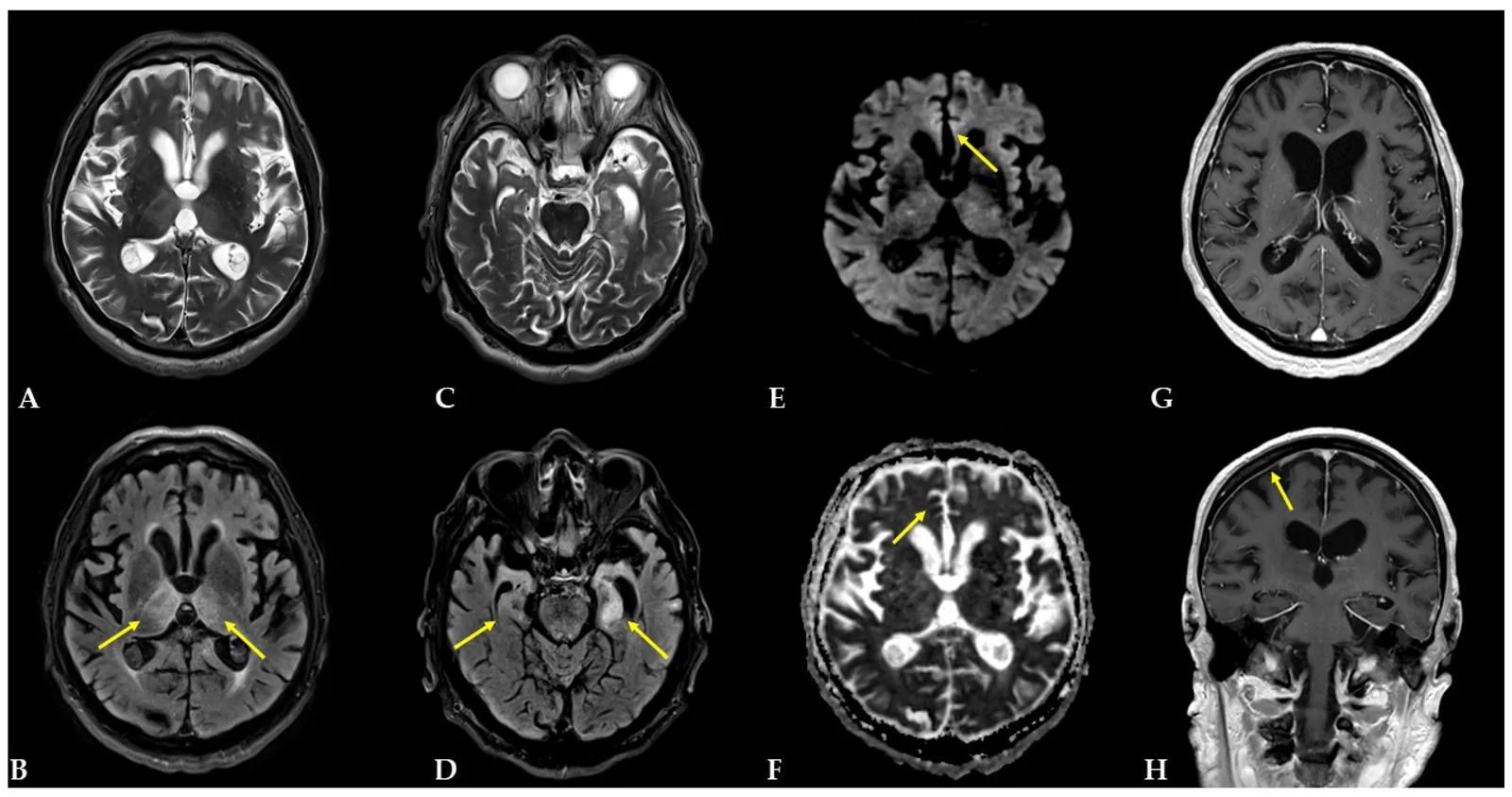
MRI images of patient 4 annotated with arrows. (**A**,**B**) Axial plane, T2W and FLAIR: asymmetric high signal intensities in both thalami; (**C**,**D**) axial plane, T2W and FLAIR: asymmetric high signal intensities in both hippocampi; (**E**,**F**) axial plane, only in DWI/ADC: abnormal signal intensities in the cortex of the frontal lobe; (**G**,**H**) axial and coronal plane, T1WCE: meningeal contrast enhancement.

Patient 4 demonstrated an asymmetric high signal intensity in both hippocampi and thalami on T2W and FLAIR images, and restricted diffusion on DWI/ADC. Only on DWI/ADC were abnormal signal intensities found in the cortex of the frontal lobe (Figure 4). A summary of the abnormal brain MRI findings in USUV patients is presented in Table S2.

### 3.2. Literature Review of Brain MRI Findings in Neuroinvasive Usutu Virus Infection

Six studies reported patients with neuroinvasive USUV disease and brain MRI, all from Europe [5,9,11,18,26,27]. The characteristics of patients with MRI findings are presented in Table 3.

## 4. Discussion

In this study, we described four patients with confirmed acute neuroinvasive USUV infection who underwent brain MRI, three of whom demonstrated abnormal imaging findings. The patients included both sexes and ranged from middle to older age. One patient was immunocompromised, although all had underlying comorbidities. Among patients with abnormal MRI findings, basal ganglia and hippocampal involvement, as well as meningeal contrast enhancement, were consistently observed. According to the disease severity scale proposed by Bogović et al. [25], the patient with a normal brain MRI had moderately severe disease, whereas all patients with abnormal MRI findings had severe disease. All four patients required ICU admission and experienced prolonged hospitalization. Despite survival in all cases, neurological recovery at discharge remained incomplete, and all patients were discharged with moderate to severe disability.

Our findings expand the limited body of literature describing brain MRI abnormalities in neuroinvasive USUV infection. Like the patient reported by Santini et al. [9], our patient with a normal brain MRI experienced moderate to severe disability despite the absence of detectable neuroradiological abnormalities. In contrast, other previously reported patients with normal brain MRI findings recovered without significant neurological sequelae [11,26]. Notably, all patients with abnormal MRI findings in our cohort demonstrated meningeal contrast enhancement, a feature not previously described in published cases of neuroinvasive USUV infection. Furthermore, all patients with abnormal MRI findings showed uni- or bilateral hippocampal involvement. This observation is particularly interesting, given the report by Graninger et al. [18], which described signal abnormalities in the hippocampus associated with severe neurological disability. Other reported MRI abnormalities have involved the substantia nigra, subcortical regions [5], and the thalami, cerebral peduncles, medulla oblongata, and pons [27], highlighting the heterogeneous neuroradiological presentation of neuroinvasive USUV infection. Taken together, the available evidence suggests that limbic system involvement, particularly of the hippocampus, may represent a recurrent imaging feature in a subset of patients with neuroinvasive USUV infection. However, given the very limited number of reported cases, the clinical significance of this finding remains uncertain and warrants further investigation.

When interpreting brain MRI in patients with suspected encephalitis, particularly limbic encephalitis, the main differential diagnosis includes viral and autoimmune causes [28,29]. The primary viral pathogen affecting the temporal lobes, including the limbic system, is herpes simplex virus (HSV), particularly HSV-1 [19]. Unilateral temporal lobe abnormalities, involvement of the insular cortex, and sparing of the basal ganglia are imaging features that may suggest HSV encephalitis. In contrast, bilateral and symmetric MRI abnormalities, highly confined to the medial temporal lobes, even in the absence of the autoantibodies associated with autoimmune encephalitis, may suggest autoimmune limbic encephalitis [29]. In this study, we found that USUV may also affect the limbic system and, in some cases, the basal ganglia, underscoring the importance of ruling out potential neuroinvasive infection in patients with suspected autoimmune limbic encephalitis before initiating treatment, as the therapeutic approach is fundamentally different. The limbic system involvement, post-contrast meningeal enhancement, and possible basal ganglia involvement may suggest USUV encephalitis. Given the limited characterization of the neuroradiological manifestations of human USUV infection, MRI of the spinal cord and, where clinically indicated, the entire neuroaxis may be considered in patients presenting with severe disease. Such imaging may provide valuable insights into the neuroradiological spectrum of this rare infection and contribute to a better understanding of its underlying neurological involvement.

The main limitations of this study include its retrospective design and small sample size, which prevented formal statistical analysis and limited the generalizability of the findings. The small sample size may reflect both the predominantly asymptomatic course and the underreporting of the disease, as well as the historically limited nationwide availability of MRI, although access to MRI has improved in recent years. Another limitation is the inability to assess lesion evolution over time, given the lack of follow-up MRI examinations. Nevertheless, this study also has several strengths. To the best of our knowledge, this is the largest series of patients with neuroinvasive USUV infection to include standardized clinical assessment and brain MRI evaluation, enabling a systematic analysis of neuroradiological findings in relation to disease severity and functional outcomes. Therefore, this study provides important insights into MRI findings in such patients and significantly addresses the current knowledge gap regarding the neuroimaging of this rare condition.

## 5. Conclusions

Patients with neuroinvasive USUV infection and abnormal brain MRI all exhibited meningeal contrast enhancement, a finding not reported in previous studies, uni- or bilateral hippocampal involvement, suggestive of limbic encephalitis, and, in some cases, basal ganglia involvement. These observations warrant further neuroradiological investigations in this emerging infectious disease. In patients presenting with encephalitis and imaging findings of limbic system involvement, neuroinvasive USUV infection should be considered in the differential diagnosis, particularly during the arbovirus transmission season. Our results are comparable to those of Graniger et al. [18], suggesting a potential MRI pattern in patients with neuroinvasive USUV infection. It is a clinically significant recognition because patients with neuroinvasive USUV infection may present with a severe disease course, while its management differs substantially from that of autoimmune limbic encephalitis. Brain MRI in patients with neuroinvasive USUV infection may provide clinicians with additional information that could affect decision-making and the disease course. Spinal MRI may also be considered in these patients, as it could help further refine the differential diagnosis and facilitate earlier diagnostic clarification. However, this needs to be verified in studies with a larger number of participants.

## Figures and Tables

**Table 1:** Sociodemographic, clinical, and laboratory features of patients with neuroinvasive USUV infection.

	Patient 1	Patient 2	Patient 3	Patient 4
Sociodemographic features				
Sex	Male	Male	Female	Male
Age	63 years	47 years	75 years	86 years
Residency	Suburbs	Countryside	City	City
Clinical data				
Past medical history	Relapsing follicular NHL; autologous stem cell transplantation; type 2 diabetes mellitus	Sleep apnea	Hypothyroidism, extrapyramidal disorder; status post laminectomy and post-spinal fusion	Dementia; arterial hypertension; glaucoma; benign prostatic hyperplasia
Charlson comorbidity index (points)	5	0	3	5
Immunocompromised	Yes	No	No	No
Admission to the ICU	Yes	Yes	Yes	Yes
Invasive mechanical ventilation	Yes	Yes	Yes	Yes
Duration of illness before hospitalization	9 days	5 days	2 days	5 days
Duration of hospitalization	39 days	86 days	107 days	50 days
Severity of clinical symptoms (points) ^1^	22	27	29	27
Vital status at discharge	Alive	Alive	Alive	Alive
mRS (points) at discharge	4	4	5	5
Blood/CSF laboratory parameters				
L (3.4–9.7 × 10^9^/L)	6.1	6.1	8.6	14.3
CRP (<5 mg/L)	32.9	19.7	86.2	18.8
CSF cells (N/μL)	60	2000	288	70
CSF proteins (0.17–0.37 g/L)	1.68	1.57	0.7	1.11
CSF glucose (2.5–4.44 mmol/L)	4.1	3.7	2.9	3.8
CSF neutrophils (%)	1	4	28	16
CSF lymphocytes (%)	88	61	50	74

ICU—intensive care unit; mRS—modified Rankin scale; NHL—non-Hodgkin lymphoma; L—leukocyte count; CRP—C-reactive protein; CSF—cerebrospinal fluid; ^1^ according to Bogović et al. [25].

**Table 2:** Virology results of patients with neuroinvasive USUV infection.

Method	Patient 1	Patient 2	Patient 3	Patient 4
CSF RT-PCR	Negative	Positive	Positive	Positive
Urine RT-PCR	Positive	Negative	Negative	Negative
Serum ELISA	Positive	Negative	Negative	Positive
CSF ELISA	Negative	Negative	Negative	Negative
Serum VNT	Positive (1:40)	NT	NT	NT
Sequencing	USUV Europe 2	USUV Europe 2	USUV Europe 2	-

CSF = cerebrospinal fluid; RT-PCR = reverse transcriptase-polymerase chain reaction; ELISA = enzyme-linked immunosorbent assay; VNT = virus neutralization test; USUV = Usutu virus; NT = not tested.

**Table 3:** Studies reporting patients with human neuroinvasive USUV infection and with brain MRI features.

Study	Country	N Patients with Neuroinvasive USUV Infection	N Patients with Brain MRI	Patient Characteristics	Brain MRI Findings	Outcome
Pecorari et al., 2009 [5]	Italy	1	1	Female in her 60s, diffuse large B-cell lymphoma	Abnormal signal intensities in the substantia nigra and parietal/frontal subcortical regions	Slight neurological disability
Santini et al., 2015 [9]	Croatia	3	1	Female, 29 years, previously healthy	Normal	Moderate disability
Simonin et al., 2018 [11]	France	1	1	Male, 39 years, previously healthy	Normal	No significant disability
Pacenti et al., 2019 [26]	Italy	8	1	Male, late 60s, multiple comorbidities including malignancy (not specified)	Normal	Complete recovery
Graninger et al., 2022 [18]	Austria	1	1	Male, 81 years, with previously known left-sided hemifacial spasms and bilateral deafness	Initial MRI normal; follow-up MRI showed left hippocampal hyperintensity (FLAIR)	Severe disability
Szabó et al., 2025 [27]	Hungary	1	1	Female, 61 years, history of malignancy (ampullary adenocarcinoma)	Symmetric T2 hyperintensities involving the thalami, cerebral peduncles, medulla oblongata, and pons, with parenchymal microhaemorrhages	Death

## Data Availability

Data is contained within the article, and further inquiries can be directed to the corresponding author.

## Supplementary Materials

The following supporting information can be downloaded at: https://www.mdpi.com/article/10.3390/diagnostics16203269/s1: Table S1: Evaluation of disease severity in patients with neuroinvasive USUV infection according to Bogović et al. [25]; Table S2: Brain MRI findings in patients with neuroinvasive USUV infection.
