# Supplementary material for: Brain MRI Features of Human Neuroinvasive Usutu Virus Infections
Source: Diagnostics (Basel). 2026 Oct 9;16(20):3269. doi: 10.3390/diagnostics16203269 (PMC13703486; doi:10.3390/diagnostics16203269)
Supplement: Supplementary file 1 [file diagnostics-16-03269-s001.zip › diagnostics-4572227-supplementary.pdf]

Table S1. Evaluation of disease severity in patients with neuroinvasive USUV infection, according to Bogović et al. [25].

Present symptom/sign	Duration (days)	Points[1]
Headache	1-5	1
	>5	2
Fever[2]	1-3	1
	4-7	2
	>7	3
Vomiting	1-3	1
	>3	2
Meningeal signs	1-5	1
	>5	2
Tremor	/	7
Pareses	/	
1-2 extremities		7
>2 extremities or trunk		9
Urine retention	/	5
Cognitive function disturbance	/	5
Conscious disturbance	/	
Somnolence		3
Stupor/coma		7
Therapy for elevated intracranial pressure[3]	1-5 doses	3
	>5 doses	5

/ not applicable; [1] Zero points if an individual symptom/sign was absent; [2] >37.5°C; [3] 250 mL 20% manitol followed by 20 mg furosemide parenterally (up to two times daily)

Table S2. Brain MRI findings in patients with neuroinvasive USUV infection.

N	Normal/ abnormal MRI	Abnormal MRI findings (MRI sequences)	Interval between hospital admission and MRI (days)	Interval between onset of symptoms and MRI (days)
Patient 1	Normal	None	1	10
Patient 2	Abnormal	Asymmetric hyperintensity of both thalami, the entire right temporal lobe (including hippocampus), and cerebellum (T2W and FLAIR), with meningeal enhancement	3	8
Patient 3	Abnormal	Asymmetric hyperintensity of both hippocampi and symmetric hyperintensity in cerebellar hemispheres (T2W and FLAIR), restricted diffusion of hippocampi and cerebellum (DWI/ADC), and meningeal enhancement	4	6
Patient 4	Abnormal	Asymmetric hyperintensity of both hippocampi and thalami (T2W and FLAIR), restricted diffusion of hippocampi and thalami (DWI/ADC), isolated restricted diffusion of frontal lobe cortex (DWI/ADC), and meningeal enhancement	10	15

T2W – T2-weighted images; FLAIR – Fluid attenuated inversion recovery; DWI/ADC – Diffusion-weighted images/Apparent diffusion coefficient
